# The emerging roles of GPRC5A in diseases

**DOI:** 10.18632/oncoscience.104

**Published:** 2014-11-25

**Authors:** Honglei Zhou, Isidore Rigoutsos

**Affiliations:** ^1^ Computational Medicine Center, Jefferson Alumni Hall, Thomas Jefferson University, Philadelphia, PA

**Keywords:** *GPRC5A*, *RAI3*, tumor suppressor, oncogene, dual-behavior, cancer

## Abstract

The ‘Retinoic Acid-Inducible G-protein-coupled receptors’ or RAIG are a group comprising the four orphan receptors GPRC5A, GPRC5B, GPRC5C and GPRC5D. As the name implies, their expression is induced by retinoic acid but beyond that very little is known about their function. In recent years, one member, GPRC5A, has been receiving increasing attention as it was shown to play important roles in human cancers. As a matter of fact, dysregulation of GPRC5A has been associated with several cancers including lung cancer, breast cancer, colorectal cancer, and pancreatic cancer. Here we review the current state of knowledge about the heterogeneity and evolution of GPRC5A, its regulation, its molecular functions, and its involvement in human disease.

## INTRODUCTION

**Figure 1:**
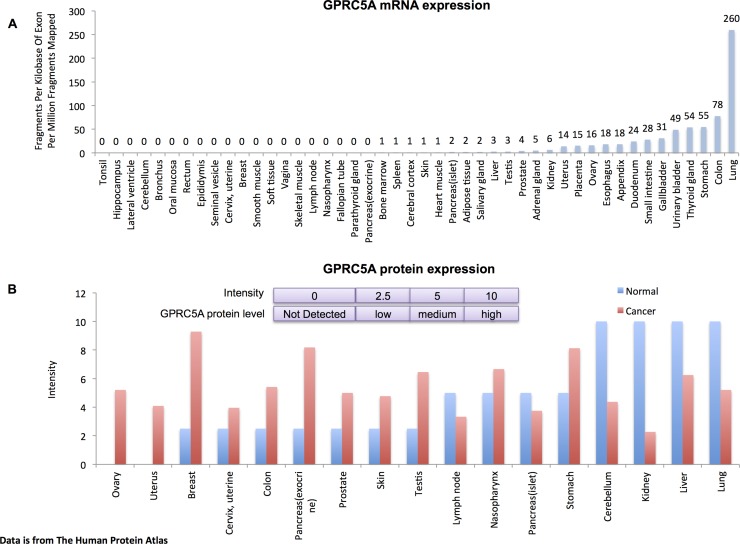
*GPRC5A* expression A. Levels of expression of *mRNA* in different organs and tissues. mRNA expression is measured by using RNA-seq. The expression levels are plotted as number of Fragments Per Kilobase of Exon Per Million Fragments Mapped. B. Expression of *protein* in different organs and tissues in both normal and cancer. Protein levels are measured by using immunohistochemistry. Different intensities indicate different expression levels. The Figure was compiled using data is from The Human Protein Atlas http://proteinatlas.org/.

The G protein-coupled receptor, class C, group 5, member A (*GPRC5A*), also known as Retinoic acid-induced gene 3 (*RAI3*) or Retinoic acid-induced gene 1 (*RAIG1*) was first cloned in 1998 [1]. *GPRC5A* is primarily expressed in lung and lowly expressed or absent in other tissues (Figure 1A). In normal tissues the GPRC5A protein mainly locates at plasma membrane, perinuclear vesicle, endoplasmic reticulum, Golgi apparatus and extracellular vesicular exosomes [1-5] – see also the Human Protein Atlas at http://www.proteinatlas.org. Previous *GPRC5A* studies indicated that it plays important roles and that its dysregulation could result in many different types of cancer in humans (Figure 1B) as well as other diseases [5-13]. To better understand the field of *GPRC5A* research, here we summarize and discuss what is currently known about this gene and its protein.

### Heterogeneity and Evolution of *GPRC5A* gene

**Figure 2:**
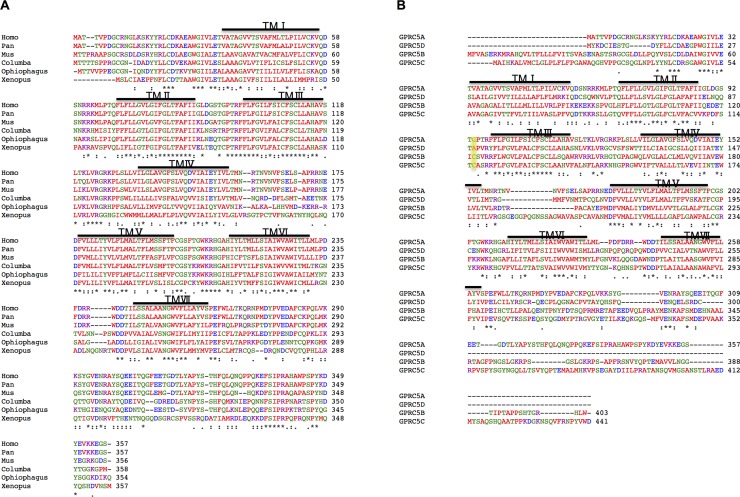
**A.** Alignment of human GPRC5A across several species. Identical (*) and conservatively substituted (:) amino acids are indicated. Gaps are shown with “-” whereas putative TM segments are over-lined. B. Alignment of the four receptors from the human class C group 5. Identical (*) and conservatively substituted (:) amino acids are indicated. Gaps are shown with “-” whereas putative TM segments are over-lined.

*GPRC5A* is expressed in different mammals and other tetrapods as well as in birds, reptiles, amphibians, and fish [14]. Phylogenetic analysis shows that the amino acid sequence of human GPRC5A shares a high percentage of sequence identity with orthologues from other species, indicating that it has been conserved in evolution: 99% with *Pan troglodytes*, 76% with *Mus musculus*, 55% with *Columba livia*, 52% with *Ophiophagus Hannah*, and 46% with *Xenopus laevis*. See also Figure 2A. In addition to GPRC5A, there are three more members in this group of receptors, each with different degrees of evolutionary conservation. Amino acid sequence alignment of the four members shows that GPRC5A shares 31%~42% sequence identity with the other three members [15, 16] – see also Figure 2B. The four members of group 5 receptors have high sequence identity within the transmembrane segments while the major difference occurs at N-terminus. The N-termini of GPRC5A and GPRC5D are shorter than those of GPRC5B and GPRC5C. On the other hand, both GPRC5B and GPRC5C contain highly conserved cysteines in the first and second extracellular loops (highlighted in Figure 2B), but GPRC5A and GPRC5D lack this residue [15]. In the absence of a long N-terminus, it's unlikely that GPRC5A can bind an agonist at its amino-terminus domain. Instead, agonists may bind to the 7 transmembrane (TM) of GPRC5A, GPRC5B, GPRC5C and GPRC5D, similarly to class A GPCRs [15, 17, 18]. Currently, no ligand is known for any of the members of RAIG. The human *GPRC5A* gene contains many Single Nucleotide Polymorphisms (SNPs) in its 5′ untranslated regions (5′UTR), its coding region, and its 3′UTR (listed in Additional file 1): whether these variants have any functional impact (through post-transcriptional effects or changes in the amino acid sequence) is not currently known.

### Transcriptional Regulation of *GPRC5A*

**Figure 3:**
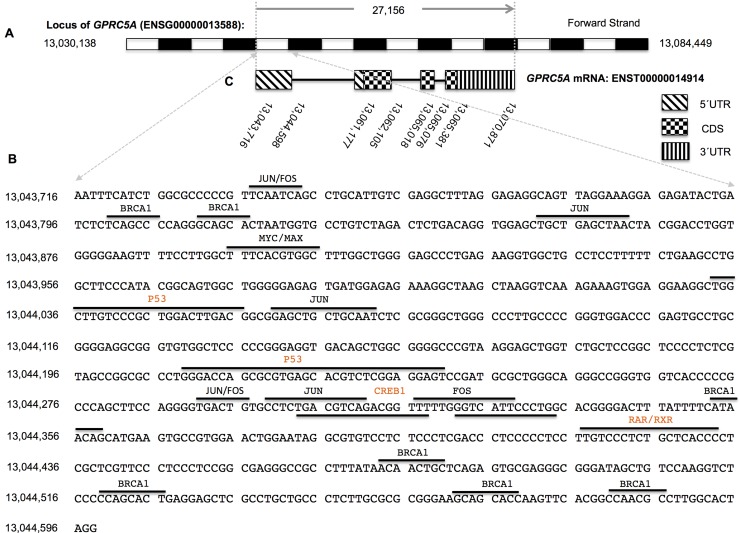
Chromosomal locus, gene architecture and transcription factor binding information A. *GPRC5A* gene (ENSG00000013588) location is chromosome 12:13,030,138-13,084,449. B. RAR/RXR binding site, CREB binding site, TP53 binding sites, BRCA1 binding sites, FOS/JUN binding sites, and MYC binding site are located between 13,043,716 and 13,044,596. C. *GPRC5A* mRNA (ENST00000014914) is spliced from the region between 13,043,716 and 13,070,871. Transcription factor information was compiled using data from the ENCODE project predictions from JASPAR [92].

**Figure 4:**
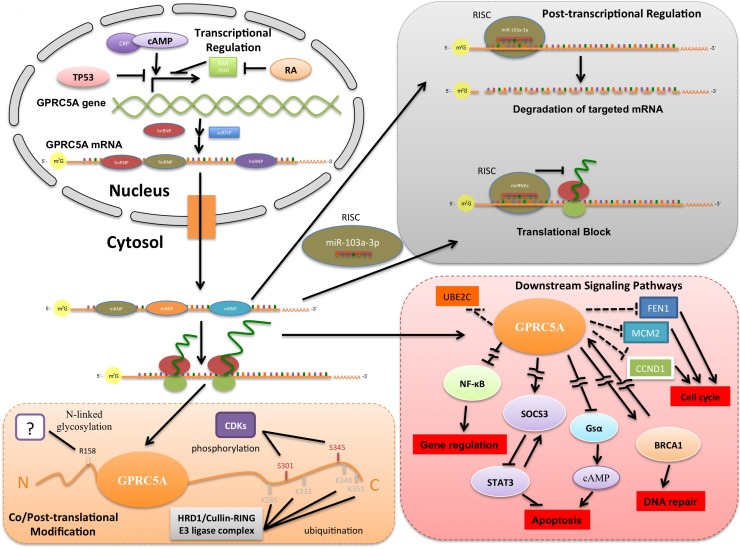
A systemic view of GPRC5A's activity In the nucleus, TP53 inhibits *GPRC5A* gene transcription by binding to *GPRC5A*'s promoter region. On the other hand, cAMP promotes *GPRC5A*'s transcription by binding to the latter's promoter region along with cAMP receptor protein (CRP). RA binding to RAR/RXRs results in dissociation of co-repressor and recruitment of co-activator proteins that in turn promotes *GPRC5A* gene transcription. In the cytosol, *GPRC5A* mRNA is targeted by miR-103a-3p, resulting mainly in mRNA degradation. GPRC5A protein is phosphorylated at S301 and S345 during mitosis by cyclin-dependent kinases. In addition, R158 in GPRC5A is likely glycosylated *in vivo*. Also, L285, L333, L348 and L353 at the C-terminus of GPRC5A might be ubiquitinated by the HRD1/Cullin-Ring E3 ligase complex. With regard to downstream signaling pathways, GPRC5A could promote SOCS3 and BRCA1 expression while inhibiting NF-κB and Gsα expression, but details of this mechanism are lacking. GPRC5A may also inhibit UBE2C, FEN1, MCM2 and CCND1 expression based on previously reported indirect evidence [64]. Arrows in this diagram represent up-regulation or activation. T-joints indicate down-regulation or inhibition (direct evidence) whereas *dashed* T-joints indicates the available evidence is indirect. Interrupted lines indicate a connection that involves one or more intermediate pathways.

*GPRC5A* was first identified as a retinoic acid-induced gene and its locus contains a novel retinoic acid response element (RARE) at its proximal 5′ upstream region (Figure 3) [19]. In the absence of retinoic acid, retinoic acid receptors bind to the RARE as RAR/RXR heterodimers [20, 21], recruiting co-repressor proteins and repressing *GPRC5A* gene transcription. Binding of agonist ligands to RAR/RXRs results in dissociation of co-repressor and recruitment of co-activator proteins, which in turn promotes *GPRC5A* gene transcription (Figure 4) [19, 22-26].

The GPRC5A gene locus also contains p53 consensus DNA binding sequences in the promoter region (Figure 3 and Figure 4) [27]. Overexpression of wild-type p53 represses *GPRC5A* expression in 2774qw1 human ovarian tumor cell line [27]. Both microarray and quantitative RT-PCR in MDA-MB-468, BT-20, BT-549, and SK-BR-3 cells which contain mutant p53 and in T47D, MCF7, ZR-75-1, and BT474 cells which contain wild type p53 show that *GPRC5A* mRNA is up-regulated in p53 mutated cell lines [27]. Besides p53 and retinoid acid, *GPRC5A* could be regulated by cAMP as there is a cAMP-responsive element (CRE) in its promoter region and *GPRC5A*'s expression could be induced by cAMP signaling (Figure 3 and Figure 4). Interestingly, cAMP and retinoic acid might synergistically regulate GPRC5A expression [28]. In addition to these three regulatory elements in promoter region, the ENCODE project has identified several additional transcription factors that bind to *GPRC5A* promoter region (Figure 3 and Additional file 2 and http://genome.ucsc.edu/).

The GPRC5A gene locus contains multiple BRCA1 binding sites (Figure 3). BRCA1 could work either as an activator or as a repressor by association with different other transcription factors [13, 29-37]. A previous study showed that knockdown of BRCA1 results in down-regulation of *GPRC5A* expression [13], suggesting that BRCA1 may work as a positive activator of *GPRC5A* expression. The locus also contains FOS and JUN binding sites (Figure 3). FOS and JUN are encoded by proto-oncogenes and bind to each other to form the Activator Protein-1 (AP-1) complex, which could in turn activate gene transcription [38-40]. AP-1 activity is usually induced by stimulations such as growth factors, pro-inflammatory cytokines and is UV-radiation-dependent on the MAPK cascade, JNK and p38 [38, 41-43]. As *GPRC5A* expression could be induced by serum in cell culture [27, 44], it is very likely that FOS and JUN binding sites within *GPRC5A* gene are functional.

Another transcription factor MYC also binds to *GPRC5A* promoter region in some cancer cells like HeLa and HepG2 cells (Figure 3), whereas not in embryonic stem (ES) cells [45]. *GPRC5A* expression level is lower in ES cells than trophoblast and overexpression of MYC in ES cells down-regulate GPRC5A mRNA [45].

### Post-transcriptional Regulation of *GPRC5A*

MicroRNAs (miRNAs) are small non-coding RNAs that function as transcriptional and post-transcriptional regulators of gene expression [46] and would be among the first candidates to post-transcriptionally regulate GPRC5A. However, very little is currently known in this regard. In a recent report, *GPRC5A* mRNA was shown to be targeted at its 5′UTR by miR-103a-3p in pancreatic cells [47]. This is a very interesting finding considering that currently only a handful of examples are known in the literature where targeting of a 5′UTR by a miRNA leads to the down-regulation of the respective mRNA (Figure 4). In this particular case, it was shown that overexpression of miR-103a-3p reduces both *GPRC5A* mRNA and protein in cells. In addition to miR-103a-3p, computational predictions (Table 1) using the *rna22* algorithm [48, 49] suggest that many other putative miRNA target sites are present along the length of GPRC5A's mRNA.

**Table 1:** Number of distinct miRBase miRNAs and target sites that *rna22* predicts target *GPRC5A* (P-val ≤ 0.05)

Region	Number of targeting miRNAs (predicted)	T Number of argeting sites (predicted)
5′UTR	343	98
CDS	595	223
3′UTR	1170	922

Among the important post-transcriptional regulators are also RNA binding proteins (RBPs) [50] as well as long non-coding RNAs (lncRNAs) [51-54]. Both are suspected of playing roles in post-transcriptionally regulating *GPRC5A* but no data are available at this time [55-57].

### Molecular function of *GPRC5A*

As stated above, GPRC5A's short N terminus makes it unlikely that it binds ligands through that region [15]. Chimeric receptors with the N-terminus of the metabotropic glutamate receptor 1 (mGluR1) and the 7 TM region of GPRC5A could bind mGluR1 ligands [40], but is not activated [15]. This indicates that the signaling transduction cascade may differ in the case of GPRC5A from other class C GPCRs. Interestingly, overexpression of GPRC5A in Nthy-ori 3-1 cells down-regulated Gsα expression and reduced intracellular cAMP levels compared to cells transfected with the empty vector [28]. In turn, cAMP signaling could mediate *GPRC5A* expression by cAMP-responsive element (CRE) motif close to *GPRC5A* gene's transcription initiation site [28]. The results indicate GPRC5A may form a feedback loop in regulating cAMP signaling.

In addition to cAMP signaling, GPRC5A is also involved in the NF-κB and STAT3 signaling pathways [58, 59] – see also Figure 4. Knockout of *GPRC5A* in mouse lung cells inhibits *SOCS3* expression, which leads to persistent STAT3 activation under stimulation and up-regulation of STAT3-regulated cell survival genes, resulting in cell transformation and resistance to cell death [58, 60-62]. Besides, in *GPRC5A* knockout mouse lung cells, both basal NF-κB activation and lipopolysaccharide-induced NF-κB activation are elevated [59]. Enhanced NF-B activation up-regulates multiple NF-κB target genes in cells, leading to increased inflammation [42, 59, 63].

Besides STAT3 and NF-κB signaling pathway, GPRC5A is involved in regulating the cell cycle. It probably achieves this through *FEN1, MCM2, CCND1* and *UBE2C* as the expression of these genes is up-regulated following loss of GPRC5A expression in lung adenocarcinoma [64]. But a detailed understanding of the regulatory mechanism is still lacking.

The GPRC5A protein is also post-translationally modified. For example, it is known that the amino acid residues S301 and S345 are phosphorylated during mitosis [65-68] – see also Figure 4. Phosphorylation of GPRC5A may play a role during cell cycle progression. Besides phosphorylation, N-linked glycosylation probably occurs at R158. Recent studies show that GPRC5A could also be ubiquitinated at K285, K333, K348 and K353 [69-74], but detailed mechanisms and functional roles of GPRC5A ubiquitination are not yet available.

GPRC5A also interacts with multiple proteins *in vivo*. Among the probable interacting partners of GPRC5A are EIF4A1 and HSPA9 [75]. In addition, it has been shown that GPRC5A is co-fractionated with GTF2F2, MPV17, NOLC1, and SLC25A3 under certain experimental conditions. In other experiments, GPRC5A was shown to interact with TRIM69 or UBC [69-74, 76, 77].

### *GPRC5A* and its elucidated roles in diseases

We next review the current body of literature regarding the involvement of *GPRC5A* in various diseases (Table 2).

**Table 2:** Dysregulation of GPRC5A in different diseases

Disease	Level Cancer vs. Normal	Methods	Reference
Oral Squamous Cell Carcinoma	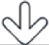	IHC	[10]
Non-Small Cell Lung Carcinoma	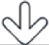	Microarray; qRT-PCR;	[78, 85]
Chronic Obstructive Pulmonary Disease	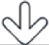	Microarray; qRT-PCR;	[85]
Breast Carcinoma^#^	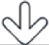	NGS; RT-PCR	[13]
Primary Myelodysplastic Syndeome	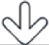	Microarray	[86]
Hepatocellular Carcinoma	N/A^(d)^	Microarray	[8]
Hepatocellular Carcinoma	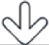	qRT-PCR	[84]
Colorectal Adenocarcinoma	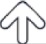	LC-MS/MS	[12]
Breast Carcinoma (Primary)	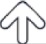	Microarray	[81]
Breast Carcinoma (Invasive)	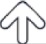	CPA; IHC	[82]
Colorectal Adenocarcinoma	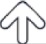	LC-MS/MS;IHC	[5]
Atherosclerosis^(a)^	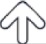	Microarray	[9]
Gastric Carcinoma	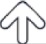	Microarray; qRT-PCR;	[7]
Breast Carcinoma	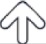	qRT-PCR	[80]
Intreheptic Cholangiocarcinoma	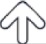	Microarray; qRT-PCR	[11]
Pancreatic Ductal Adenocarcinoma^(b)^	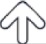	Microarray	[27]
Breast Carcinoma^(c)^	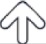	Microarray; qRT-PCR	[27]
Hepatocellular Carcinoma	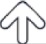	qRT-PCR; WB; IHC	[83]

# *BRCA1 5382insC* mutation carrier vs. *BRCA1* wild-type patients.

(a) The SMC cell line is analyzed in this study.

(b) Pancreatic cell lines are analyzed in this study.

(c) Breast cell lines are analyzed in this study.

(d) *GPRC5A* expression level is compared within patients before treatment and after treatment. *GPRC5A* is up-regulated after treatment.

- ***GPRC5A* and non-small cell lung carcinoma:** In non-small cell lung carcinoma (NSCLC), *GPRC5A* mRNA levels are lower than in adjacent normal tissues [78]. Ectopic overexpression of GPRC5A in NSCLC cell line H1792 reduces cells' growth in soft agar. In addition, homozygous *GPRC5A* knockout mice are much more prone to develop lung tumors at 1-2 years of age than heterozygous or wild type mice. But deficiency of GPRC5A does not cause significant developmental defects in the lungs of mice [79]. Other parallel studies have shown that knockout of *GPRC5A* in mice leads to activation of NF-κB, promoting lung inflammation and tumorigenesis, and enhances the transformed phenotype in normal and malignant lung epithelial cells through STAT3 signaling pathway [58, 59]. Studies also show that lung tumorigenesis in the *GPRC5A*-KO mouse model is augmented by nicotine-derived nitrosamine ketone (NNK) and that gene expression changes are induced by tobacco carcinogens [64].
- ***GPRC5A* and oral squamous cell carcinoma**: *GPRC5A* expression level is very high in normal oral tissue, especially in differentiated areas whereas in oral squamous cell carcinoma (OSCC) its expression is repressed [10]. In OSCC, *GPRC5A* expression is negatively correlated to OSCC's differential level. *In vitro* experiments revealed that overexpression of GPRC5A in OSCC CAL27 cells suppresses the cells' anchorage-independent growth activity, indicating that *GPRC5A* plays a tumor suppressor role in oral tissue.
- ***GPRC5A* and breast cancer**: The evidence so far regarding *GPRC5A*'s role in breast cancer has been conflicting. In one breast cancer study [80] it was shown that *GPRC5A* mRNA is up-regulated in 19 of 25 primary breast cancers and in 6 of 11 breast cancer cell lines examined, compared with normal mammary gland tissue. Moreover, knockdown of GPRC5A by small interfering RNA (siRNA) in breast cancer cell lines MCF7 and T47D suppressed cancer cells' growth [80]. Another group found that in breast cancer, the expression of *GPRC5A* along with that of *PYCARD* and *FXYD3* can serve as a good predictor of treatment outcome and enhances the predictive power of tumor size [81]. However, another study [82] reported that although *GPRC5A* expression is up-regulated in breast cancer, its expression level is not associated with tumor stage, lymph node status, histological grading or histological tumor type, or with overall and recurrence-free survival. Another recent study showed that the germline inactivating mutation c.183delG of *GPRC5A* is enriched in breast cancer patients carrying the *5382insC* allele of *BRCA1* [13]. Lastly, *in vitro* experiments revealed that GPRC5A is involved in forming radiation-induced BRCA1 and RAD51 DNA repair foci and that knockdown of GPRC5A attenuates DNA repair foci in cells following radiation [13].
- ***GPRC5A* and colorectal cancer**: In normal colon tissue, *GPRC5A* is expressed at low levels [1]. The majority of colon epithelium is negative or weak for GPRC5A expression, while only neuroendocrine cells within the colonic crypts show high expression level on plasma membrane [5]. In colorectal cancer, *GPRC5A* is abundantly present in tumor epithelium with the neuroendocrine cells showing strong staining on the plasma membrane [5]. Within neoplastic epithelium, its localization pattern is variable with most colorectal cancer tissues displaying diffuse cytoplasmic expression. Strong cytoplasmic expression of GPRC5A in colorectal cancer tissues is significantly associated with disease recurrence in Dukes' A-C (stage 1-3) patients when compared to low or negative expression of GPRC5A in cancer tissues. In another study, 33 cases of primary colorectal cancer and 16 colon polyps were subjected to LC-MS/MS analysis [12]. In these samples, GPRC5A level is higher in both metastatic and non-metastatic colorectal cancer than it is in polyps indicating that GPRC5A could be used as a biomarker in the diagnosis of colorectal cancer.
- ***GPRC5A* and gastric cancer**: In normal small intestine, *GPRC5A* is expressed at very low levels whereas in gastric cancer tissues *GPRC5A* is elevated significantly [7]. The pattern of *GPRC5A* expression in gastric cancer tissues is quite different from that of normal mucosa and of adjacent noncancerous lesion samples. This raises the possibility that *GPRC5A* can be used as a potential biomarker and a treatment target for gastric cancer based on its membrane localization and its association with cancer cell proliferation.
- ***GPRC5A* and hepatocellular carcinoma**: Based on the currently available evidence it is not clear whether in liver *GPRC5A* acts an oncogene or as tumor suppressor. In one study, it was reported that *GPRC5A* expression is higher in hepatocellular carcinoma (HCC) than in para-tumor or in normal liver tissues [83]. Higher expression of *GPRC5A* is associated with lower overall and disease-free survival rate in HCC patients [83]. However, in another study researchers reported that *GPRC5A* mRNA levels are lower in seven newly established cell lines from patient derived tumor xenografts [84]. Yet another study showed that *GPRC5A* mRNA levels are higher in HCC patients after 8-week treatment with peretinoin and that the expression of 233 genes including *GPRC5A* could classify patients into one of two groups, recurrence vs. non-recurrence, with a prediction accuracy rate of 79.6% [8].
- ***GPRC5A* and other diseases**: In chronic obstructive pulmonary disease (COPD) patients, the levels of GPRC5A protein were significantly lower in normal bronchial epithelia (NBE) compared with healthy controls [85]. As the levels of *GPRC5A* mRNA decrease from normal people to patients with either COPD or adenocarcinoma it is likely that in this context *GPRC5A* acts as a tumor suppressor. On the other hand, in intrahepatic cholangiocarcinoma (ICC), *GPRC5A* mRNA levels are higher than in normal tissue [11] suggesting that they could serve as a novel biomarker for classifying and diagnosing this highly fatal type of carcinoma. In myelodysplastic syndrome (MDS), *GPRC5A* mRNA levels were found to be lower in CD34+ cells, which could explain these cells' susceptibility to cell damage [86].

## CONCLUSION

The *GPRC5A* gene is conserved from mammals to fish [14]. In the disease context, *GPRC5A* was originally reported as a tumor suppressor in non-small cell lung carcinoma. Later its tumor suppressor ability was also shown in oral squamous cell carcinoma. Subsequent reports indicated that in breast cancer, colorectal cancer and pancreatic cancer *GPRC5A* could also behave as an oncogene. This dual behavior makes *GPRC5A* a very interesting gene to study. Nonetheless, its likely interactions with many other factors some of which may be present in some cell types and absent in other cell types suggest that a lot more research work will be required to understand how this dual behavior arises. Recent research efforts revealed that mRNA transcripts could play additional important roles, besides their protein coding ability, by decoying miRNAs [87-91]. As the *GPRC5A*'s mRNA contains many putative miRNA-binding sites it is conceivable that some of its functional roles are effected through the decoying of one or more miRNAs. To prove this hypothesis and to elucidate the regulatory roles of *GPRC5A* mRNA and protein additional research is needed.

Not surprisingly considering that GPRC5A was originally identified as a retinoid acid induced molecule, *GPRC5A* contains a RAR/RXR binding site [1]. In addition, *GPRC5A*'s expression is suppressed by p53 and mutation of p53 leads to increased levels of *GPRC5A* in p53-mutant breast cancer cells [27]. Furthermore, as the GPRC5A locus is favored by multiple other transcription factors such as BRCA1, FOS, JUN and MYC, dysregulation of one or more of these transcription factors may lead to abnormal expression of GPRC5A [13, 29, 38, 45].

With respect to post-transcriptional regulation, we recently reported that miR-103a-3p can regulate GPRC5A protein expression by targeting the 5′UTR of *GPRC5A*'s mRNA. This finding suggests that to understand *GPRC5A*'s post-transcriptional regulation one needs to consider miRNA targets beyond its mRNA's 3′UTR. Moreover, RBPs and possibly lncRNAs might also be involved in regulating *GPRC5A*. However, no such interactions are currently known.

With respect to post-translational modification, several articles report that GPRC5A protein is phosphorylated at S301 and S345 during mitosis [65-68]. N-glycosylation and uniquitination also occur but the physiological functions of these modifications remain unclear.

With respect to its participation in signaling pathways relatively little information is currently available. One line of work reported that *GPRC5A* expression impacts on the cAMP signaling pathway [28]. In another line of work it was shown that knocking out GPRC5A leads to the activation of the NF-κB and STAT3 signaling pathways and results in cell proliferation and resistance to cell death [58, 59]. Additionally, loss of GPRC5A could result in up-regulation of *FEN1, MCM2, CCND1* and *UBE2C*, all of who are involved in cell cycle regulation [64]. However, it is still unknown how exactly GPRC5A interacts these pathways.

Some of the previous studies attempted to find a potential ligand for GPRC5A [15, 28, 58] but without success. In addition, it is unclear how GPRC5A is activated. As this gene is found to be dysregulated in many cancers we expect that further studies will provide additional insights about its post-transcriptional and post-translational regulation and function. In turn these findings will help advance the diagnosis and eventually the treatment of cancers through the manipulation of key stages of this protein's lifecycle.

## SUPPLEMENTARY TABLES




